# A high-penetrance intergenic variant at 9p21 confers melanoma susceptibility

**DOI:** 10.21203/rs.3.rs-9636010/v1

**Published:** 2026-05-12

**Authors:** Linh T Bui-Raborn, Lorenza Pastorino, Donato Calista, Phuc H Hoang, Jessica L Scales, Mai Xu, Sophie Papiernik, Xiaoyu Wang, Bruna Dalmasso, Rohit Thakur, Stefania Pellegrini, Joshuah Yon, Timothy Myers, Chia-Han Lee, Jacob Williams, Haoyu Zhang, Tongwu Zhang, William Bruno, Zaida García-Casado, Enrica Teresa Tanda, Francesco Spagnolo, Monia Di Prete, Irene Bottillo, Siranoush Manukian, Maria Chiara Scaini, Katerina P Kypreou, Lucia Di Nardo, Miriam Potrony, Paula Aguilera-Peiró, Nesreen Shahrour, Tam-Anh Tran, Yao Yu, Chad D Huff, Paul Scheet, Lee E Wheless, Rebecca I Hartman, Jia Liu, Weiyin Zhou, Wen Luo, Aurélie Vogt, Kristine M Jones, Belynda D Hicks, Monica Rodolfo, Irene Stefanaki, Cristina Pellegrini, Carlo Cota, Chiara Menin, Maria Concetta Fargnoli, Ketty Peris, Susana Puig, Eduardo Nagore, Paola Grammatico, Italian Melanoma Intergroup, The MelaNostrum Consortium, Jianxin Shi, Paola Ghiorzo, Kevin M Brown, Maria Teresa Landi

**Affiliations:** 1 Laboratory of Translational Genomics, Division of Cancer Epidemiology and Genetics, National Cancer Institute, Rockville, MD, USA; 2 Department of Internal Medicine and Medical Specialties, University of Genoa, Genoa, Italy; 3 Cancer Genetics, IRCCS Azienda Ospedaliera Metropolitana, Genoa, Italy; 4 Department of Dermatology, Maurizio Bufalini Hospital, Cesena, Italy; 5 Integrative Tumor Epidemiology Branch, Division of Cancer Epidemiology and Genetics, National Cancer Institute, Rockville, MD, USA; 6 Biostatistics Branch, Division of Cancer Epidemiology and Genetics, National Cancer Institute, Rockville, MD, USA; 7 Immunology and Molecular Oncology Unit, Veneto Institute of Oncology IOV-IRCCS, Padua, Italy; 8 Laboratory of Genetic Susceptibility, Division of Cancer Epidemiology and Genetics, National Cancer Institute, Rockville, MD, USA; 9 Cancer Genomics Research Laboratory, Leidos Biomedical Research, Frederick National Laboratory for Cancer Research, Frederick, MD, USA; 10 Laboratory of Molecular Biology, Instituto Valenciano de Oncología, València, Spain; 11 Medical Oncology 2, IRCCS Azienda Ospedaliera Metropolitana, Genoa, Italy; 12 Department of Surgical Sciences and Integrated Diagnostics, University of Genoa, Genoa, Italy; 13 Dermatopathology Research Unit, San Gallicano Dermatological Institute IRCCS, Rome, Italy; 14 Laboratory of Medical Genetics, Experimental Medicine Department, San Camillo-Forlanini Hospital, Sapienza University, Rome, Italy; 15 Unit of Medical Genetics, Department of Medical Oncology and Hematology, Fondazione IRCCS Istituto Nazionale dei Tumori, Milan, Italy; 16 1st University Clinic of Dermatological and Venereal Diseases, Andreas Syggros Hospital, School of Medicine, National and Kapodistrian University of Athens, Athens, Greece; 17 Immunology Research Core Facility - Gemelli Science and Technology Park (GSTeP), Fondazione Policlinico Universitario Agostino Gemelli IRCCS, Rome, Italy; 18 Dermatologia, Dipartimento Universitario di Medicina e Chirurgia Traslazionale, Università Cattolica del Sacro Cuore, Rome, Italy; 19 Biochemistry and Molecular Genetics Department, Hospital Clínic of Barcelona, IDIBAPS, Barcelona, Spain; 20 CIBER of rare diseases (CIBERER), Instituto de Salud Carlos III, Barcelona, Spain; 21 Dermatology, Hospital Clínic de Barcelona, Barcelona, Spain; 22 Department of Epidemiology, Division of Cancer Prevention and Population Sciences, The University of Texas MD Anderson Cancer Center, Houston, TX, USA; 23 Department of Dermatology, Vanderbilt University Medical Center, Nashville, TN, USA; 24 Department of Medicine, Division of Epidemiology, Vanderbilt University Medical Center, Nashville, TN, USA; 25 Dermatology Section, Veterans Affairs Boston Healthcare System, Jamaica Plain, MA, USA; 26 Department of Dermatology, Brigham and Women's Hospital, Boston, MA, USA; 27 Harvard Medical School, Boston, MA, USA; 28 Division of Cancer Epidemiology and Genetics, National Cancer Institute, Bethesda, MD, USA; 29 Unit of Translational Immunology, Department of Experimental Oncology, Fondazione IRCCS Istituto Nazionale dei Tumori, Milan, Italy; 30 Department of Biotechnological and Applied Clinical Sciences, University of L'Aquila, L'Aquila, Italy; 31 U.O.C. Dermatologia, Dipartimento di Scienze Mediche e Chirurgiche, Fondazione Policlinico Universitario A. Gemelli IRCCS, Rome, Italy; 32 Dermatology, Instituto Valenciano de Oncología, València, Spain; 33 School of Medicine, Universidad Católica de Valencia San Vicente Mártir, València, Spain; 34 Experimental Medicine Department, Laboratory of Medical Genetics, Sapienza University of Rome, Rome, Italy

**Keywords:** cutaneous melanoma, family study, genetic susceptibility, deletion, structural variant, founder mutation, *CDKN2A*

## Abstract

Approximately 10% of cutaneous malignant melanoma cases are familial. Variants in *CDKN2A* account for up to 40% of melanoma-prone families, with an additional ~10% explained by other genes. Many *CDKN2A* mutation-negative families show linkage to chromosome-band 9p21, which harbors *CDKN2A*, suggesting non-coding variants may contribute to familial risk. Here, whole-genome sequencing revealed a novel 100 kb deletion mapping to 9p21 in a gene-desert region, 205 kb from *CDKN2A,* cosegregating in a four-case family from Genoa, Italy. The deletion overlaps melanocyte enhancers that interact with the promoters of *CDKN2A* p16 and p14 transcripts and is predicted to reduce p16 expression. Using a nearby rare exonic variant in *MTAP* (rs755147810) on the deletion haplotype, we searched for deletion carriers in WES data from high-risk melanoma patients and controls and identified 22 cases and a single control carrying rs755147810 and the deletion. The association with melanoma in case-control analysis was highly significant (3,319 cases and 5,680 controls; *P*=1.27x10^−6^; OR=27.50). We observe loss-of-heterozygosity of the wild-type allele in a carrier’s tumor sample. The founder haplotype with the most recent common ancestor dates approximately 26 generations, broadly overlapping the period when Italy was struck by devastating outbreaks of plague that decimated the population creating a genetic bottleneck. Our results provide evidence of a high-penetrance intergenic variant conferring melanoma susceptibility, with potential for genetic screening of high-risk individuals.

Cutaneous malignant melanoma (CMM) is the most heritable of all solid tumors ^1^, with roughly 10% of cases occurring in the context of melanoma families ^2^. Germline variants in the *CDKN2A* gene account for up to 40% of familial cases ^2-8^, while a single predisposing variant in the *CDK4* gene has been reported in a small number of CMM kindreds, representing approximately 2% of high-risk melanoma families ^3,9-11^. Variants in several other genes, including a mutation in the promoter of *TERT* ^12,13^, as well as protein-coding variants in *BAP1* ^14-17^ and shelterin telomere end-protection complex members *POT1* ^18,19^, *ACD* ^20^, *TERF2IP* ^20^, and *TINF2* ^21^ collectively account for a small proportion of melanoma families. Roughly half of melanoma families, however, remain unexplained by pathogenic variants in established high-penetrance susceptibility genes. Some families may be explained by higher polygenic risk ^22,23^, although a proportion of dense multi-case families not explained by known high-penetrance mutations have low polygenic risk scores ^24^. Thus, rare protein coding mutations in undiscovered high- or medium-penetrance genes, or non-coding variation impacting high-penetrance genes could account for the missing heritability.

Notably, a proportion of the unexplained families show genetic linkage to chromosome band 9p21, the locus harboring *CDKN2A*, but nonetheless lack protein-coding *CDKN2A* variants ^25,26^. While 9p21 harbors another established tumor suppressor gene in melanoma, *CDKN2B* ^27^, multiple studies have failed to find pathogenic protein-coding *CDKN2B* mutations in high-risk melanoma families ^25,26,28^, suggesting that these families may be explained by non-coding variation affecting *CDKN2A*. Studies of intronic variation ^29^ or copy number changes within or over *CDKN2A* ^30-32^ have yielded findings for only a handful of families, suggesting the presence of undiscovered variants altering *CDKN2A* function in high-risk families.

Here, as a part of a larger study of melanoma susceptibility in Mediterranean populations, we performed germline whole-genome sequencing (WGS) in a single melanoma case from a four-case melanoma pedigree from Genoa, Italy and identified a novel 100 kb deletion in a gene desert region on chromosome band 9p21 located between the *CDKN2B-AS1* and *DMRTA1* genes. The 9p21 deletion co-segregates with melanoma in the pedigree. Using whole exome sequencing from high-risk melanoma patients and controls, we confirmed the deletion in 22 melanoma cases and one single control, all from Italy. Our study demonstrates, for the first time, a non-coding intergenic structural variant as a high-penetrance susceptibility variant for melanoma. The founder mutation has been estimated to have occurred around the 14^th^ century.

## RESULTS

### A novel 100 kb deletion on chromosome band 9p21

**Fig. 1:**
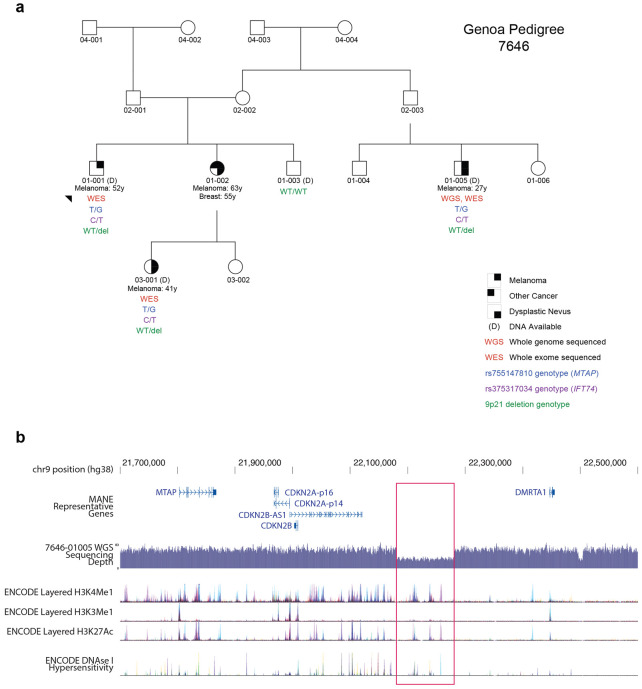
Identification of a germline 100 kb deletion in a high-risk melanoma pedigree. **a,** Pedigree of a four-case melanoma family from Genoa, Italy (Family 7646). DNA was available for three cases (01-001, 01-005, and 03-001). Diagnoses of melanoma, dysplastic nevi, and other cancers are noted along with age of onset for cancer cases. One available case (01-005) was subjected to whole-genome sequencing (WGS), while all three had whole-exome sequencing (WES) data generated; family member 01-003 was genotyped via a PCR-based assay. Genotype for rare variants in *MTAP* (rs755147810) and *IFT74* (rs375317034) as well as the 100 kb non-coding deletion (9p21 deletion) on chromosome band 9p21 are noted, showing cosegregation with melanoma in the pedigree. **b,** WGS identified a ~100 kb deletion (outlined in red and indicated by lower WGS sequencing depth) in individual 7646-01005 approximately 200 kb downstream of *CDKN2A*. Layered histone marks and DNAse I hypersensitivity sequencing data for multiple cell lines profiled by ENCODE is shown, with several prominent regulatory peaks contained within the deletion region.

We performed WGS of the proband from a four-case family from Genoa Italy (Fig. 1a, Family ID 7646, individual 01-005) with the goal of identifying structural variants (SVs) near or within known susceptibility genes. Two of the other cases from this pedigree (01-001 and 03-001) were subjected to whole-exome sequencing (WES). Melanoma cases within this pedigree were characterized by relatively high numbers of melanocytic nevi (all cases as well as an unaffected family member had > 50 nevi each, **Supplementary Table 1**). Three of the four cases in the pedigree (01-002, 01-005 and 03-001) also had dysplastic nevi, two of the four cases had multiple primary melanomas, and two had relatively early age of onset (27, 41, 52, and 63 years old at diagnosis; Fig. 1a, **Supplementary Table 1**).

Consistent with prior clinical screening performed in this family, we did not observe any protein-coding variants in *CDKN2A*, nor did we observe the *CDKN2A* intronic splicing mutation previously reported by Harland and colleagues ^29^ in any of the sequenced family members. No rare protein-coding variants were found in other established melanoma susceptibility genes, nor was the patient a carrier of the p.E318K variant of *MITF* or the *TERT* promoter mutation previously reported in melanoma families ^12,13^. In the WGS data from individual 01-005, we did, however, identify a heterozygous 100,767 bp deletion (ClinSV coordinates chr9:22,180,210 - 22,280,977; hg38) on chromosome band 9p21 located approximately 205 kb downstream of *CDKN2A* (Fig. 1b, **Supplementary Table 2**) in a gene desert region between *CDKN2B-AS1* and *DMRTA1*. This region does not overlap any annotated protein-coding genes but contains two smaller regions harboring prominent H3K4Me1 and H3K27Ac marks in multiple cell types assessed by ENCODE, suggesting a likely gene regulatory function. We did observe additional structural variants (Fig. 1b, **Supplementary Table 2**), including a 7.8 kb heterozygous region of copy loss (chr9:22,496,528-22,504,345) downstream of *DMRTA1*, however this variant is common (deletion allele frequency of 0.7) in the Medical Genome Reference Bank (MGRB) dataset ^33^. No structural variants were evident directly over *CDKN2A*, nor other nearby gene coding-regions. Using the Database of Genomic Variants (DGV) ^34^, which includes structural variants from the Genome Aggregation Database (gnomAD), we determined the 100 kb deletion observed in sample 7646-01005 is novel. We note a few SVs that overlap the deleted region (**Supplementary Table 3**), the majority observed in only a single individual.

We confirmed the presence of this 9p21 deletion and assessed whether it cosegregates with melanoma in the pedigree using a multiplexed PCR assay. We confirmed the presence of this deletion in melanoma case 7646-01005 as well as the two additional melanoma cases in this family (7646-01001 and 7646-03001), while an unaffected member (7646-01003) lacked this variant (Fig. 1a, **Supplementary Fig. 1**), confirming cosegregation of the deletion and linkage to 9p21. Sanger sequencing of the PCR product specific to the deletion allele clarified the boundaries from that derived from WGS of 7646-01005 (chr9:22,180,207-22,280,977; coordinates are left-aligned). This family did not carry rare protein-coding variants in the other prominent tumor suppressor gene in the 9p21 region, *CDKN2B* (**Supplementary Table 4**). We did, however, observe a rare missense variant in the *MTAP* gene on 9p21 (rs755147810; p.Ser3Ala), predicted as benign by SIFT ^35^, PolyPhen2 ^36^, MutationAssessor ^37^, CADD ^38^, LRT ^39^, and AlphaMissense ^40^, but damaging by MetaLR ^41^ (**Supplementary Table 4**).

### Identification of the deletion variant in additional high-risk melanoma patients

We next assessed whether this deletion is carried by other melanoma-prone individuals by assessing a larger whole exome case-control study (WES) performed by the MelaNostrum Consortium ^42^ consisting of Italian, Spanish, and Greek melanoma cases and controls (3,574 cases, 2,673 controls; **Supplementary Table 5, Supplementary Fig. 2**). All cases had a high-risk phenotype: most had a family history (with multiple affected members sequenced in some families), while the remainder were sporadic cases with multiple primary melanomas and/or early onset (<37 years). Given that this deletion itself is not reliably detectable from WES data, we first sought to identify one or more rare proxy variants from exome sequencing data marking a haplotype carrying the deletion. Specifically, using WES data from two additional members of the Genoa pedigree 7646 (01-001 and 03-001; Fig. 1a), we identified two variants in the region that met the criteria of being rare (MAF <1 x 10^-4^, gnomAD v.4, Non-Finnish European) and cosegregating with melanoma in the pedigree: rs755147810 in the *MTAP* gene (chr9: 21,802,755:T:G; p.Ser3Ala for multiple *MTAP* transcripts; 377 kb upstream of the deletion); and rs375317034 (chr9:26,980,644:C:T in an intron of the *IFT74* gene; 4.7 Mb downstream of the deletion; **Supplementary Table 4**).

We then assessed these proxy markers in the WES data from melanoma cases and controls and observed rs755147810 (*MTAP*) in 17 additional cases (not counting three cases from pedigree 7646) and only a single control, all from Italy, and mostly from Genoa (**Supplementary Table 1, Supplementary Table 6**). Of the cases, 14 had a family history from 12 total multi-case families (**Supplementary Fig. 3**), as well as three patients with multiple primary melanoma diagnoses (ages 21, 31, and 49 at first diagnosis, respectively). rs375317034 (*IFT74*) was found in three additional cases and no controls; of these, two cases did not carry rs755147810 (**Supplementary Table 6**). We directly genotyped all carriers of rs755147810/*MTAP* and rs375317034/*IFT74* for the 9p21 deletion as well as DNAs from any available additional relatives from families that were not part of the WES study using a multiplex PCR assay. All carriers of the *MTAP* variant also harbored the deletion, while the two individuals that carry only the *IFT74* variant did not (**Supplementary Fig. 1**), suggesting that only rs755147810 is in strong linkage disequilibrium and a specific proxy for the deletion. Sanger sequencing confirmed an identical breakpoint in all carriers.

Besides the carriers identified from our WES dataset, we were able to separately confirm the presence of both the deletion and *MTAP* rs755147810 variants in two additional cases for which DNA was available (6143-02002 and 7793-03001, **Supplementary Fig. 1, Supplementary Figs. 3j** and **3l**), as well as one family member with a dysplastic nevus diagnosis (3914-03002, **Supplementary Fig. 3a**). In total, we identified 22 melanoma cases and one control harboring the deletion (**Supplementary Fig. 2**). Beyond the index pedigree (pedigree 7646; 3/3 available cases), we were able to confirm the presence of the deletion in all available cases from the families with identified carriers (Cesena pedigree 3914, 2/2 cases, **Supplementary Fig. 3a**; Genoa pedigree 7793, 2/2 cases, **Supplementary Fig. 3l**; Genoa pedigree 6143, 2/2 cases; **Supplementary Fig. 3j**) and Genoa pedigree 7816, 2/2 cases, **Supplementary Fig. 3k**), consistent with the deletion being a high-penetrance, cosegregating familial melanoma risk variant. Similar to what is observed for pedigree 7646, the larger set of cases tended to have a high number of melanocytic nevi, multiple diagnoses of dysplastic nevi, and an early age of onset (**Supplementary Table 1**). Although our WES samples could theoretically include additional carriers of the deletion haplotype with recombination between the deletion and rs755147810, this is unlikely due to their close genomic proximity.

### Variant association with melanoma risk

We formally tested for association of the *MTAP* rs755147810 proxy variant with melanoma risk across unrelated individuals within the entire WES dataset. Where multiple members of a family had been sequenced, we retained only the family individual with the earliest age of onset for testing. We further excluded samples with less than 10 sequencing reads over rs755147810; in total we assessed 3,219 cases and 2,266 controls (**Supplementary Fig. 2, Supplementary Table 5**). Across the larger dataset of cases and controls from Italy, Spain, and Greece, the association of the *MTAP* variant with melanoma was highly significant with an odds-ratio consistent with that expected for a high-penetrance variant (*P* = 0.0015; OR = 11.31; **Supplementary Table 7**). Considering that this variant was only observed in samples from Italy, we also tested this association within the smaller subset of Italian WES data, with the association remaining highly significant (*P* = 0.0028; OR = 10.20; **Supplementary Table 7**). We further noted that most samples we identified harboring the deletion and rs755147810 were collected in Genoa, Italy. We therefore directly genotyped an additional 514 healthy controls from Genoa for both rs755147810 and the deletion itself, finding no carriers of either. Combining these controls into the larger testing, the association in the fully Italian/Spanish/Greek (*P* = 3.90 x 10^−4^; OR = 13.88; **Supplementary Table 7**) and Italian (*P* = 3.69 x 10^−4^; OR = 14.01; **Supplementary Table 7**) samples remained significant, as was a test of samples specifically from Genoa (*P* = 0.0073; OR = 8.63; **Supplementary Table 7**). Finally, to further increase power, we assessed the frequency of this variant in an additional 3,000 individuals from hospital-based WES data from Italy (100 melanoma cases and 2,900 individuals investigated for a number of other diseases; see Methods), none of whom carried rs755147810. Combining these additional Italian samples with the melanoma case-control WES data and additional controls from Genoa, the association was again strongly significant for the full dataset (Italian/Spanish/Greek: 8,999 individuals, 3,319 cases and 5,680 controls; *P* = 1.27 x 10^−6^; OR = 27.50) and also for all Italian samples combined (*P* = 1.50 x 10^−7^; OR = 33.97; **Supplementary Table 7**).

### The 9p21 deletion region interacts with the promoters of *CDKN2A*, *CDKN2B*, *MTAP*

**Fig. 2:**
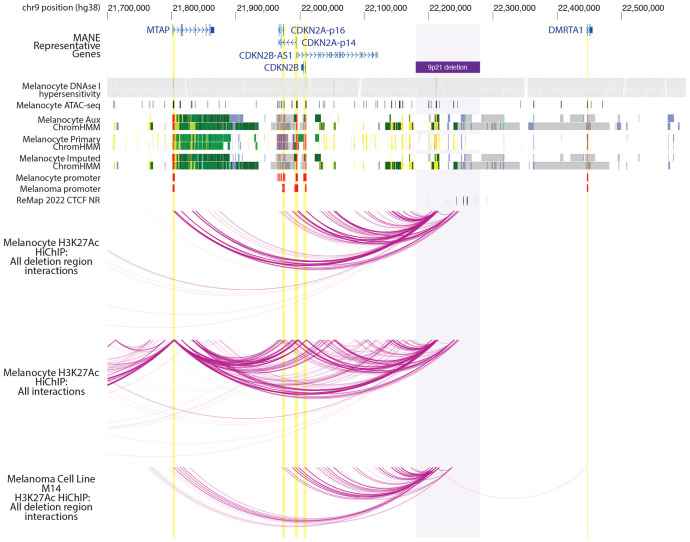
Chromatin interactions between regulatory regions within the 9p21 deletion and the promoters of *CDKN2A*, *CDKN2B*, and *MTAP*. The 100 kb deletion (highlighted in purple, “9p21 deletion”) does not overlap with protein-coding genes but contains annotated melanocyte enhancers (Epigenome Roadmap ChromHMM data, data from two melanocyte cultures shown) and regions of open chromatin (Epigenome Roadmap DNAse I hypersensitivity sequencing, two melanocyte cultures shown; melanocyte ATAC-seq peaks, the union of peaks from five cultures is shown). We note several CTCF ChIP-seq peaks located within the deletion region as annotated by ReMap 2022 ^47^. H3K27Ac Hi-ChIP peak-to-peak interactions from three melanocyte cultures are shown, with a filtered set of interactions from/to the deletion region shown in the upper looping panel and all interactions across the region shown in the lower panel. We observe loops from the deletion region to the promoters of *CDKN2A* (p16 and p14), *CDKN2B, CDKN2B-AS1*, and *MTAP* (highlighted in yellow). The lower looping panel shows all chromatin interactions to and from the deletion region in publicly-available data from the melanoma cell line M14, with observed interactions with the promoters of *CDKN2A* (p16 and p14), *CDKN2B*, *CDKN2B-AS1*, *MTAP*, and *DMRTA1* (data downloaded from the Loop Catalog ^45^)

**Fig. 3:**
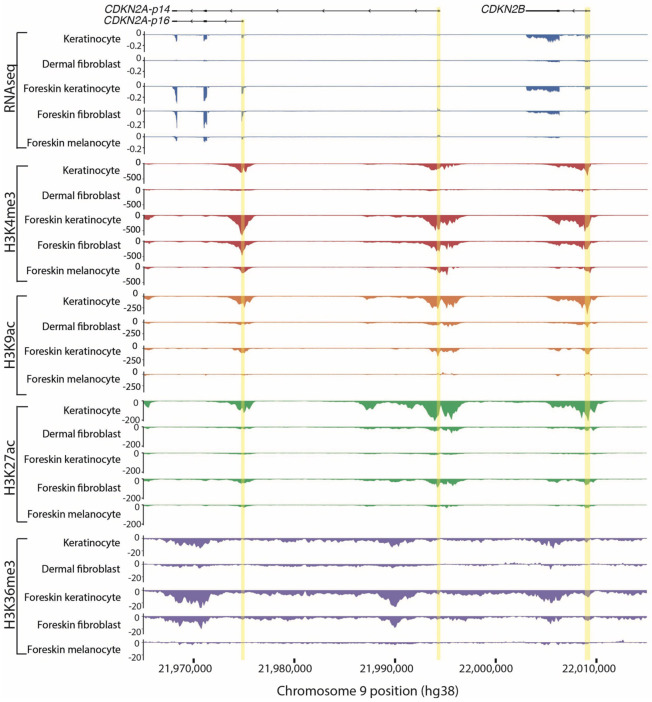
Predicted transcriptional and epigenome effects of the 9p21 deletion. AlphaGenome predictions of the effects of the 9p21 deletion on transcription (RNA-seq) and histone marks (histone ChIP-seq) over the *CDKN2A* and *CDKN2B* genes. Model predictions indicate reduced expression of all three exons of the *CDKN2A* p16 transcript as well as *CDKN2B*, accompanied by decreased promoter-associated chromatin marks (H3K4me3 and H3K9ac), reduced promoter/enhancer activity (H3K27ac), and diminished transcription across gene bodies (H3K36me3). Promoter regions for p16, p14, and *CDKN2B* are highlighted in yellow. The y-axis represents the difference between predicted signal (RNA-seq: predicted transcriptional coverage, histone ChIP-seq: predicted histone mark enrichment) for the alternative and reference alleles (ALT – REF, *e.g.* reduced expression/histone mark enrichment is reflected by negative values). RNA-seq data were derived from polyA plus RNA-seq assays for all cell types, except for dermal fibroblast (total RNA-seq assay).

The 9p21 deletion appears to harbor conserved sequence elements that could be gene regulatory and/or mediate chromatin interaction, including melanocyte enhancer elements and repressive marks (ChromHMM data from the RoadMap Epigenome Project ^43^) as well as regions of open chromatin (assessed by ATAC-seq ^44^) in human primary melanocytes (Fig. 2). To verify this hypothesis, we directly assessed interactions in the 9p21 region between regulatory regions marked by H3K27Ac in cultured human primary melanocytes. We used Hi-ChIP (High-throughput Chromatin conformation capture followed by Chromatin Immuno-Precipitation) in melanocyte cultures from three unrelated individuals of European ancestry (three technical replicates per culture, Methods) and identified significant interactions between distant regions marked by H3K27Ac ChIP-seq peaks (peak-to-peak loops). Interactions from the deletion region stem primarily from two regions (approximately chr9:22,204,000-22,217,766 and chr9:22,237,601-22,245,401; hg38) harboring both annotated enhancer elements and regions of open chromatin in melanocytes (Fig. 2). We observe direct chromatin interactions between these elements and promoters of nearby genes, including *MTAP*, *CDKN2B*, and critically both the p16 and p14 transcripts of *CDKN2A* (Fig. 2 and **Supplementary Table 8**). To further confirm chromatin interactions between deletion region with promoters of nearby genes, we used publicly available H3K27Ac HiChIP datasets from the Loop Catalog ^45^, focusing on the 16 datasets with the highest sequence coverage. 13 of these 16 datasets show chromatin interactions originating from the deletion region with eight showing interactions with promoters of nearby genes (**Supplementary Table 9**). Interactions were observed with *MTAP* (9/16), *CDKN2B* (10/16), *DMRTA1* (4/16), and critically the p14 (8/16) and p16 (5/16) transcripts of *CDKN2A*. Notably, interactions were observed to p14 and p16 promoters in the melanoma cell line M14 (**Supplementary Table 9**, Fig. 2). These data indicate that the deletion harbors regulatory elements physically interacting with *CDKN2A* promoter elements in both melanoma cells as well as melanocytes, the cell type of origin for melanoma. Such regulatory interactions are expected to be absent on chromosomes carrying the deletion. To investigate the functional consequences of the 9p21 deletion, we applied the deep learning model AlphaGenome ^46^ to predict regulatory effects on nearby genes using cell types with both histone mark and transcriptomics data (Fig. 3) and all skin-related cell and tissue types with transcriptomics data (**Supplementary Fig. 4**). Predictions were performed across a ~500 kb window overlapping *CDKN2A* and *CDKN2B* for both reference and deletion alleles, incorporating multiple cell types and regulatory modalities, including transcription (RNA-seq) and histone marks (H3K4Me3, H3K9Ac, H3K27Ac and H3K36Me3). The model predicted reduced expression of all exons of *CDKN2A* p16 and *CDKN2B* and accompanied by diminished transcription across gene bodies (H3K36Me3), and decreased promoter and enhancer-associated chromatin marks (H3K4Me3, H3K9Ac, H3K27Ac; Fig. 3, **Supplementary Fig. 4**). A modest increase in expression of *CDKN2A* exon 1A specifically associated with the p14 transcript was also predicted (**Supplementary Fig. 4**), suggesting transcript-specific regulatory effects of the 9p21 deletion on p16.

Within the melanocyte Hi-ChIP data, we also observe a lack of interactions towards the centromeric side of the deletion towards *DMRTA1*, and notably no interactions spanning the deletion itself (Fig. 2), suggesting the possibility the region harbors the boundary of a chromatin contact domain or topologically associating domain (TAD). Consistent with this hypothesis, we note multiple prominent regions of CTCF binding across multiple cell types in ChIP data from ENCODE (Fig. 2, **Supplementary Fig. 5**). Hi-C datasets from multiple different cell types clearly show patterns indicative of a contact domain with a boundary located inside the deletion region (representative examples shown in **Supplementary Fig. 5**), with chromatin regions on one side of the deletion infrequently interacting with those on the opposite side. These data suggest that in addition to a loss of enhancer elements that interact with the promoters of *CDKN2A* and other genes in the region, deletion of this region may result in loss of a prominent domain boundary, possibly allowing physical associations with distant regulatory elements that do not normally interact with these genes.

### Loss-of-heterozygosity of the 9p21 locus in a tumor sample

**Fig. 4:**
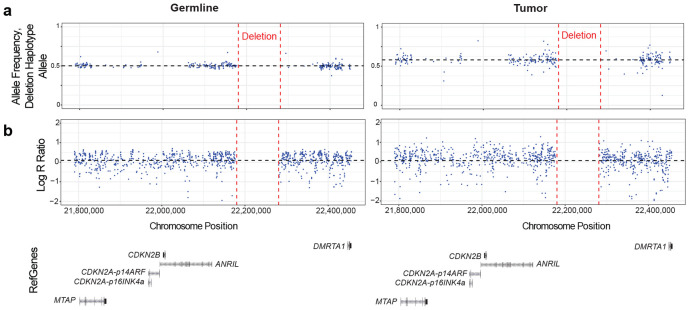
Loss of heterozygosity in the 9p21 locus in the tumor sample carrying the 9p21 deletion. **a,** Haplotype-phased allele frequency and **b,** log R ratio (LRR) plots for germline and tumor DNA samples from deletion carrier 20325-1001. Haplotype allele frequency was calculated as the proportion of the alleles on the deletion haplotype for any given heterozygous SNP. Gray dotted lines display the mean of allele frequency of (**a**) all heterozygous SNPs tested (germline sample: 0.5026, tumor sample: 0.5803), or (**b**) the mean of LRR of all SNPs (germline sample: 0.0931, tumor sample: 0.0649), vertical dotted red lines display genomic locations of the 9p21 deletion.

We assessed loss of heterozygosity (LOH) in formalin-fixed paraffin-embedded (FFPE) tumor tissue from deletion carriers using a custom capture-based sequencing assay. Specifically, we sequenced SNP positions tiled across the 9p21 region spanning from *MTAP* to *DMRTA1* and assessed loss of heterozygosity by comparing allele counts for heterozygous SNPs in both germline and tumor DNA from the same individual. While we sequenced two FFPE tumor blocks from different cases, only a single tumor (a lymphonodal metastasis sample) from family 20325 (patient 01-001) passed QC. For this sample, we assessed LOH in a haplotype-phased manner based on the genotypes for the common haplotype shared between carriers of the deletion and also with CNV-FACETS ^48^ (described below and in Methods). We observed a clear shift in allele counts consistent with loss of the wild-type allele across the entire region (Student paired t-test = 20.567, P < 2.2 x 10^−6^, Fig. 4, **Supplementary Table 10**), with the small shift consistent with the tumor purity estimate (0.3, **Supplementary Table 11**). These data are consistent with a second tumor suppressor hit targeting the wild-type (non-deletion) allele.

### The most recent common ancestor of the deletion appeared in the 14^th^ century

**Fig. 5:**
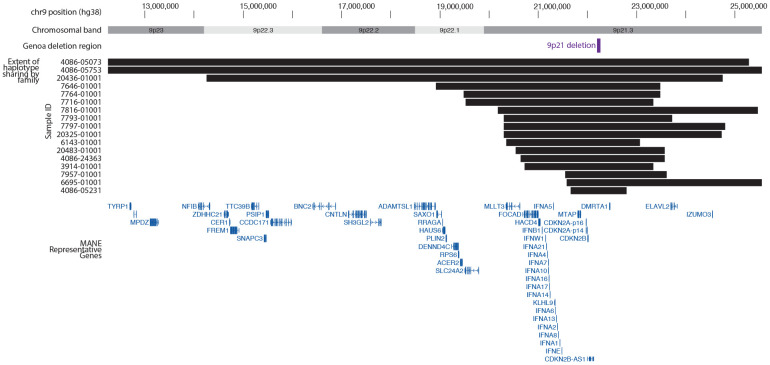
Estimated recombination breakpoints of shared haplotype in deletion carriers. UCSC Genome Browser view of chromosome 9 (chr9: 12,000,000-26,000,000; hg38) showing the extent of haplotype shared by 17 unrelated deletion carriers. The inferred recombination breakpoints are indicated by the end of the haplotype blocks (black bars), with each row representing an individual carrier. The longest shared haplotype is in two sporadic melanoma case carriers (4086-05073 and 4086-05753), while the control carrier (4086-05231) shares the smallest amount of haplotype relative to other carriers. Haplotype and recombination breakpoints were identified using SNP array data for chromosome 9 (see Methods).

The identical breakpoint boundaries of the deletion across all carriers, confirmed by Sanger sequencing, strongly support a single ancestral origin of this ~100 kb structural variant. To estimate the timing of this founder event, we characterized the distribution of historical recombination breakpoints surrounding the common haplotype that harbors the deletion. Analysis included 17 carriers, restricted to a single affected individual per multi-case family, who were verified to be unrelated (estimated kinship coefficient < 0.01) based on genome-wide SNP array data (Methods). For these 17 carriers, we implemented an algorithm to identify historical recombination breakpoints of the shared haplotype without explicitly phasing (Methods). Using these recombination breakpoints (Fig. 5) and modifying the statistical framework developed by Genin and colleagues ^49^ (Methods), we estimated 26 generations (SE= 4.6 generations) since the most recent common ancestor (MRCA) of all deletion-bearing chromosomes. Assuming a generation interval of 27 years ^50^, the founder mutation likely originated in the early 14th century, broadly overlapping the severe demographic contraction in northern Italy during the Black Death plague epidemics. Such a historical bottleneck may have facilitated the retention and drift of this rare founder haplotype within contemporary Italian populations.

## Discussion

Using WGS, we identified a ~100 kb deletion on chromosome band 9p21, approximately 200 kb downstream from the well-established familial melanoma susceptibility gene *CDKN2A* in a melanoma-prone family from Genoa, Italy. Through a rare proxy variant in the *MTAP* gene located on the same haplotype of the deletion, we identified an additional 22 high-risk melanoma cases harboring both the *MTAP* variant and the 9p21 deletion. We ruled out another structural variant found in this family, as it is commonly observed in European populations, as well as protein-coding variation in the established tumor suppressors in the region, *CDKN2A* and *CDKN2B*.

To our knowledge, this is the first evidence of a non-coding structural variant conferring high susceptibility to melanoma (OR = 27.50, P = 1.27 x 10^−6^ overall). Notably, Johansson and colleagues (back-to-back submission) have identified another 9p21 deletion partially overlapping our deletion region in families of English/Australian ancestry, with similar effect on *CDKN2A*. Together these findings confirm the crucial role of non-coding structural variants for melanoma susceptibility.

There is strong genetic evidence consistent with this deletion being a high-penetrance susceptibility allele: (1) perfect cosegregation of the deletion in all five families with DNA available for multiple cases; (2) significant associations of the *MTAP* proxy variant with melanoma risk with high odds ratios across all subjects (OR = 27.50 for Italy/Spain/Greece combined), the Italian population (OR = 33.97), or the population from Genoa, Italy (OR = 8.63); (3) the 9p21 deletion region harbors regulatory elements that interact with the p14 and p16 promoters; (4) loss of this region was predicted to reduce *CDKN2A* p16 expression using a deep-learning model; and (5) 9p21 loss of heterozygosity stemming from loss of the wild-type (non-deletion) allele in one tumor.

Notably, the *MTAP* variant rs755147810 could serve as a proxy for identifying additional potential deletion carriers from clinical WES or targeted sequencing data. The deletion is also easily assessable via whole-genome sequencing or using a simple multiplex PCR assay, providing an important tool for screening high-risk individuals. We noted additional rare germline structural variants overlapping this region in the Genome Aggregation Database (gnomAD) and Database of Genomic Variants (DGV) without phenotypic annotation ^34^; these variants should be considered additional potential susceptibility alleles warranting further investigation.

We cannot entirely rule out the *MTAP* proxy variant rs755147810 itself as a causal variant contributing to risk, given that it is a missense variant for the majority of *MTAP* isoforms (p.Ser3Ala). However, in tumors and melanoma cell lines, loss of heterozygosity at 9p21 frequently involves regions extending beyond *CDKN2A*, with shared regions of loss across samples exceeding *CDKN2A* in either direction ^51-56^. This has led to the hypothesis that there may be another major tumor suppressor in this region, including *MTAP*. Genome-wide association studies of melanoma have identified multiple independent common risk signals within the 9p21 region for both melanoma ^57,58^ and nevus count ^59-61^ with fine-mapped variants spanning from *MTAP* to the deletion region itself ^58,62^. The strongest of these signals for melanoma is located directly over *MTAP* ^58^, with the risk allele for this signal strongly *positively* associated with melanocyte expression of *MTAP* ^58,63^, which is inconsistent with a tumor-suppressor function. Moreover, multiple WES studies have failed to link rare protein coding variation in *MTAP* to melanoma risk in families or the general population ^18,19,64-66^.

The 9p21 deletion encompasses regions of enhancer regulatory marks, including H3K27Ac enhancers, in multiple cell types ^67^, including melanocytes. Importantly, we show that these enhancers directly interact with multiple gene promoters, critically including both p16 and p14 transcripts of *CDKN2A*. We also note interaction with annotated intragenic bivalent promoter within the *CDKN2A* gene (exons 2 and 3), and regulatory regions with both activating and repressive histone modifications thought to create a state of poised gene expression (Fig. 2). Using the deep-learning model AlphaGenome ^46^, we observed a predicted reduction in transcriptional output for all exons of p16 and *CDKN2B* with the deletion allele, accompanied by decreased promoter and enhancer activity (Fig. 3, **Supplementary Fig. 4**). In contrast, the first exon of *CDKN2A* which is specific for the p14 transcript was predicted to have increased expression across multiple cell types, suggesting transcript-specific regulatory effects of the 9p21 deletion. Finally, we note in the HiChIP data a lack of interactions with regions on opposite sides of the deletion region, suggesting an insulation of the region harboring *CDKN2A* and *CDKN2B* from more distant regulatory elements downstream of the deletion. Thus, loss of this region could plausibly result both in loss of normal regulatory interactions for *CDKN2A* or other genes in the region, and/or gain of novel regulatory interactions, collectively dysregulating expression of genes across the region.

In addition to physical interaction with *CDKN2A*, we observed interactions with another tumor suppressor shown to be involved in progression from nevus to melanoma ^27^, *CDKN2B*. While *CDKN2B* is a plausible candidate susceptibility gene, numerous studies have failed to establish *CDKN2B* as a familial melanoma gene ^15,18,19,28,64^ or establish rare variation in predisposition in the general population ^65,66^, suggesting that the deletion is more likely to function via *cis*-regulatory activity on *CDKN2A*. Further study will be required to comprehensively assess mechanisms by which the deletion impacts the expression of *CDKN2A* transcripts, as well as other genes, in turn contributing to increased risk.

Based on haplotype analyses and the geographic origins of most 9p21 deletion carriers and their parents, and considering an average of 27 years per generation ^50^, we infer that the most recent common ancestor (MRCA) of this deletion likely arose around the 14th century. These observations are consistent with a founder effect underlying the 9p21 deletion. Most cases are from the city of Genoa, in the Liguria region of Italy. Intriguingly, this is the same area where the *CDKN2A* founder pathogenic variants “G101W” and “E27X” have been identified ^68,69^.

Notably, the 14th century coincides with the initial introduction into Europe of the Black Death linked to the infection from *Yersinia pestis* ^70,71^. According to one of the earliest and most detailed contemporary descriptions of the epidemic in Northern Italy ^72^, plague-bearing Genoese vessels brought the disease directly to the ports of Genoa. Despite containment efforts by local authorities, successive epidemic waves spread across much of Europe ^73^. Between 1347 and 1352, the pandemic is estimated to have resulted in the deaths of over one-third ^74^ or even up to 50% ^75^ of the European population, probably the greatest public health disaster in recorded history ^72^. This abrupt and severe demographic contraction acted as a genetic bottleneck that was protracted for a long time, sharply reducing allelic diversity within the surviving population. Such stochastic loss of genetic variation likely facilitated the retention and propagation of the 9p21 deletion haplotype contributing to the persistence of this genetic variant in modern populations.

## Material and Methods

### MelaNostrum study cohorts:

the study cohorts come from the MelaNostrum Consortium study. As the National Cancer Institute (NCI) exclusively received deidentified samples and data from collaborating centres, NCI had no direct contact or interaction with the study participants, and did not use or generate any identifiable private information, MelaNostrum study has been classified as ‘not human subject research’ (NHSR) in accordance with the Federal Common Rule (45 CFR 46; eCFR.gov). Collaborating centers obtained informed consent for publication of human data from participants under protocols approved by their respective Institutional Review Boards. A detailed description of each study from all MelaNostrum contributing centers is in **Supplementary Note**.

### Whole genome sequencing (WGS):

Genomic DNA was extracted from blood samples using standard method. DNA was quantified using the QuantiFlour dsDNA System (Promega). DNA was diluted to 25ng/μl and underwent fragment analysis using the AmpFLSTR Identifiler PCR Amplification Kit (Thermo Fisher Scientific). Sequencing was performed by the Broad Institute (https://www.broadinstitute.org) using Illumina HiSeq X system following Illumnia-provided protocols for 2 x 151-bp paired-end sequencing. The sequence data were aligned to the human genome assembly GRCh38 using the local alignment tool bwa mem ^76^ with default parameters and the aligned BAM files were transferred to the National Institute of Health (NIH) HPC system (https://hpc.nih.gov) for downstream analyses. We used Picard tools (CollectWgsMetrics, http://broadinstitute.github.io/picard) to assess overall sequencing metrics and mosdepth ^77^ to calculate chromosome-level sequencing depth.

### Identification of structural variants from WGS data:

We used two different approaches to look for structural variants (SVs) in 9p21 locus in WGS data. First, we calculated the read depth (RD) for 9p21.3 locus using samtools depth tool ^78^ with the minimum base quality of 20 (-q 20) and minimum mapping quality of 20 (-Q 20). For each sample, scaled coverage was then calculated by dividing RD at each position with the mean RD of the whole region, and coverage plots were generated using ggplot2. The deletion was confirmed by manually assessing read coverage across the region in the samples using Integrative Genomic Viewer (IGV). Second, we performed SV-calling using ClinSV ^79^ using bam files containing reads mapped to locus 9p21.3 using default parameters. The 9p21 deletion was confirmed by both methods.

### Confirmation of deletion boundaries:

In order to confirm the exact genomic coordinates for the deletion, we performed a PCR on the same gDNA used for WGS. We used primers outside the deletion boundaries as identified on IGV or ClinSV (Fseq and Rseq, **Supplementary Table 12**). Coordinates and structure of the breakpoint were subsequently assessed via Sanger sequencing (Applied Biosystems).

### Deletion genotyping:

To confirm whether samples carrying *rs755147810* (*MTAP*) and *rs375317034* (*IFT74*) variants also carry the deletion, as well as to genotype the deletion in additional control samples from Genoa, we used a multiplex PCR assay using two primer sets. One primer set amplified across the deletion on the variant allele (F_del_ and R_del_, **Supplementary Table 12**) and a second primer set amplified the wild-type allele (F_WT_ and R_WT_, **Supplementary Table 12**). Multiplex PCR was performed using 10 mM each primer and 10ng/μl of gDNA with the following cycle: 95°C for 30 seconds, 61.8°C for 30 seconds and 72°C for 1.5 minutes for 35 cycles. PCR products were assessed using a 2% agarose gel. Amplification of the wild-type allele yields a 284 bp product and amplification of the variant allele yields a 593 bp product.

### Structural variant databases.

The DGV structural variant merged database ^34^ was downloaded as a bigbed file (https://hgdownload.soe.ucsc.edu/gbdb/hg38/dgv/), and converted to a bedfile using UCSC tool bigBedToBed ^80^. To identify structural variants that overlap with deletion coordinates, we used bedtools intersect ^81^.

### Whole exome sequencing:

Whole-exome sequencing was performed using multiple exome platforms and protocols, including previously-published data using the SeqCap EZ Human Exome Library v2.0 or v3.0 (Roche NimbleGen) ^18^; EZ Human Exome + URT Library (Roche NimbleGen) ^82^ as described previously. Additional samples were sequenced using Roche Kapa HyperExome kits. Libraries were prepared using the Kapa HyperPlus Kit using xGen Dual Index UMI Adapters (IDT, Coralville, IA) according to Kapa-provided protocol. Briefly, genomic DNA sample libraries were amplified pre-hybridization by ligation-mediated PCR using 5-7 cycles of amplification depending on input and were purified with Agencourt AMPure XP Reagent (Beckman Coulter Life Sciences, Indianapolis, IN) according to the Kapa-provided protocol. Amplified sample libraries were quantified using Quant-iT^™^ PicoGreen dsDNA Reagent. Prior to hybridization, amplified sample libraries with unique barcoded adapters were combined in equal mass amounts into 1.1 μg pools for multiplex capture. Exome sequence capture was performed with Roche Kapa HyperExome, and binding, washing, and recovery of captured DNA, as well as subsequent amplification and purification, was performed as described in Roche’s HyperCap v3 protocol. Pools of amplified captured DNA were then quantified via Roche Kapa’s Library Quantification Kit for Illumina on the LightCycler 480 (Roche, Indianapolis, IN). The resulting captured library pools were loaded on a NovaSeq 6000 (Illumina, San Diego, CA) and paired-end sequencing was performed using read lengths of 2x150bp.

Paired-end reads were aligned to the human genome assembly GRCh38 using the local alignment tool bwa mem ^76^ (with default parameters. Duplicate reads were marked using the Picard tool MarkDuplicates (http://broadinstitute.github.io/picard) and base quality of the input reads was calculated using GATK ApplyBQSR ^83^ (Broad Institute). We used three different-variant callers (Sentieon Haplotyper, Sentieon DNA Scope Model 2.0 and Illumina Strelka) ^84-86^ to call variants from the aligned bam files, only variants called by at least two callers were considered for analysis. For the scope of this study, we focused on rare variants in the 7646 family for genes located on chromosomal band 9p21 and other melanoma susceptibility genes. For variant QC, we retained variants that have a total read depth ≥ 10, read depth for alternative allele ≥3, 0.2 ≤ AB Het ≤ 0.8 for heterozygotes and genotype quality ≥ 20. Then, we selected only variants with a minor allele frequency (MAF) ≤ 0.1% in gnomAD v4.1 (Non-Finish European, WGS or WES). We identified two rare variants in 9p21 locus that cosegregated with melanoma in the pedigree: rs755147810 in the *MTAP* gene (chr9: 21,802,755:T:G; p.Ser3Ala for multiple *MTAP* transcripts; GNOMAD 4.1.0 Non-Finnish European MAF = 3.4 x 10^−6^, 4/1,179,852 exome and genome alleles; gnomAD 2.1.1 Southern European MAF = 8.8 x 10^−5^, 1/11,348 exome alleles) and rs375317034 (chr9:26,980,644:C:T in an intron of the *IFT74* gene; gnomAD 4.1.1 Non-Finnish European MAF = 7.5 x 10^−5^, 79/1,060,394 exome/genome alleles; gnomAD 2.2.1 Southern European MAF = 1.8 x 10^−4^, 2/11,114 exome alleles) (**Supplementary Table 4**). We then searched for samples carrying the *MTAP* rs755147810 variant or *IFT74* rs375317034 variant in our MelaNostrum WES dataset containing all samples that passed QC (**Supplementary Table 5, Supplementary Table 6**).

### rs755147810 (MTAP) genotyping in additional controls from Genoa for statistical testing:

to increase power for statistical testing of association between *MTAP rs755147810* variant and melanoma, we genotyped *MTAP rs755147810* variant in 514 additional controls from Genoa [see **Supplementary Note** – Genoa site for more information]. PCR was performed using a custom primer set (Fmtap and Rmtap, **Supplementary Table 12**) using 40ng of gDNA, FastStart Taq DNA Polymerase (Roche Cat. No. 04738381001), and the following cycling conditions: 95°C for 30 seconds, 60°C for 30 seconds and 72°C for 60 seconds for 35 cycles. PCR products were purified with ExoSAPIT Express reagent (ExoSAP-IT^™^ Express PCR product cleanup). Purified PCR products were then used for sequencing using BigDye Terminator v1.1 Cycle Sequencing Kit (ThermoFisher) using the following cycle: 96°C for 10 seconds, 60°C for 2 minutes for 25 cycles. Reaction products were purified before capillary electrophoresis, resuspended in 15 μl of Hi-Di^™^ Formamide, then run on a 3500 Dx Genetic Analyzer (CE-IVD, IVDR).

### MTAP rs755147810 variant look up in additional 3,000 Italian samples:

We further assessed the frequency of rs755147810 (*MTAP*) in the Italian population via lookup in a WES dataset (Illumina Exome 2.5 kit sequenced on a NovaSeq6000Dx sequencer) from the Medical Genetics Laboratory, University La Sapienza, Rome. All patients were European, coming from the center-Southern area of Italy. 78% of the samples were adults, 15% 0-18 years, and 7% prenatal cases. Of these samples, 100 were melanoma cases with a mean age of diagnosis of 55 years and all were unrelated. The remainder of samples were investigated for a number of other diseases: (34% multisystemic rare diseases; 23% cardiovascular diseases, mostly cardiomyopathies; 15% neurological diseases, mostly intellectual disabilities; 9% hemochromatosis; 9% connective tissue disorders; 5% metabolic diseases; 3% cancer susceptibility; 2% autoimmune diseases).

### Testing the association of MTAP variant rs755147810 with melanoma:

We performed separate one-sided Fisher’s exact tests to examine whether the frequency of *MTAP* rs755147810 carriers were higher in melanoma cases than that in controls in the full WES dataset and in the subsets restricted to individuals from Italy or Genoa (**Supplementary Table 7**). Only samples with at least 10 sequencing reads covering the *MTAP* variant rs755147810 were included in the association analyses. Because most carriers originated from Genoa, we performed principal component analysis (PCA) to assess whether the Italian controls were genetically similar to samples from Genoa. PCA was conducted in PLINK v1.9 using imputed SNP array data from the same samples, after filtering variants for MAF > 0.01 and imputation *R*^2^ > 0.995. The PCA showed that samples from Genoa overlapped closely with the broader Italian control set, supporting the use of the Italian controls in association analyses (**Supplementary Fig. 6**).

### Genomic feature annotation of the deletion:

we used Roadmap project chromHMM data from two primary melanocyte cultures ^44^ to classify genomic regions as enhancers or promoters. Specifically, enhancers were defined using the following chromHMM states (primary: Enh, EnhG, EnhBiv; auxiliary: EnhG1, EnhG2, EnhA1, EnhA2, EnhWk, EnhBiv; and imputed: TxEnh5, TxEnh3, TxEnhW, EnhA1, EnhA2, EnhAF, EnhW1, EnhW2, EnhAC) and DNAseI hypersensitive data were used to mark active transcription. Melanocyte ATAC-seq data were generated previously by our lab from five independent melanocyte cultures ^62^, and melanoma ATAC-seq data were analysed using the publicly available omni-ATAC-seq data from nine melanoma cell lines derived from patient biopsies ^87^. Melanocyte and melanoma promoter data were generated using published imputed ChromHMM as PromU, PromD1, PromD2, TssA, PromP, PromBiv and TX_Reg for melanocytes ^43,67^, and 1_TssA, 2_PromWkD, 3_TssWkP for melanoma cell lines ^88^.

### CTCF ChIP-seq data analysis:

We annotated CTCF-bound regions using data from ReMap2022 ^47^ (https://remap.univ-amu.fr/download_page, accessed Nov 17 2025). Specifically, we downloaded the merged, non-redundant peaks for all CTCF ChIP-seq experiments (https://remap.univ-amu.fr/download_page#:~:text=hg38-,hg38,-CTCF ) and filtered to keep only peaks in chromosome 9 overlapping deletion region (**Supplementary Table 13**) using bedtools intersect tool ^81^.

### Cell culture:

Frozen aliquots of melanocytes isolated from foreskin healthy newborn males, mainly of European descent, were obtained from the Specialized Programs of Research Excellence (SPORE) in Skin Cancer Specimen Resource Core at Yale University following an established protocol. Melanocytes were grown in Dermal Cell Basal Medium (American Type Culture Collection/ATCC PCS-200-030) supplemented with Melanocyte Growth Kit (ATCC PCS-200-041) and 1% amphotericin B/penicillin/streptomycin (120-096-711, Quality Biological) at 37°C with 5% CO2. All cells tested negative for mycoplasma contamination via MycoAlert PLUS Mycoplasma Detection Kit (LT07-710, Lonza).

### H3K27ac-HiChIP assay:

We generated HiChIP data from three human melanocyte cultures from unrelated individuals (C68, C136 and C174; three biological replicates per culture). HiChIP libraries were generated using the Arima-HiC+ kit (Arima Genomics) and the Accel-NGS 2S plus DNA library Kit (Swift Biosciences) following the manufacturer's protocol. In addition, to facilitate analysis of differential interactions, we mixed a defined ratio of fixed mouse 3T3 cells with human cells ^89^. Briefly, 3.5 million human cells were crosslinked and mixed with 1M fixed 3T3 cells, enzyme-digested, biotin-labelled, and ligated. The ligated DNA was then fragmented by sonication, followed by chromatin precipitation. We tested two H3K27Ac antibodies for both specificity and sensitivity by Chromatin Immunoprecipitation coupled with quantitative Polymerase Chain Reaction (ChIP-PCR). We designed the ChIP-PCR primers to test a positive control region near the *ERBB3* promoter region and a negative non-acetylated region as a negative control (both defined using data from the Roadmap H3K27Ac ChIPSeq track from penis foreskin melanocyte primary cells). Compared to the Active Motif antibody (Cat# 39113, an H3K27Ac antibody) used by ENCODE, the H3K27Ac antibody from Cell Signaling (Cat# 8173) showed less enrichment in the negative control region (background), while it resulted in a stronger signal for the peak region near *ERBB3*; thus, all IP was performed using the Cell Signaling antibody. ChIP-PCR was performed for all samples prior to sequencing library preparation as a quality control step.

Pulled-down chromatin was reverse-crosslinked and DNA was size-selected by Ampure beads. The HiChIP product was then enriched for biotin-labelled fragments prior to sequencing library preparation. The biotin-enriched DNA fragments were end repaired and ligated to adaptors, followed by PCR amplification using KAPA library amplification kit. During the process of HiChIP procedures and sequencing library preparation, the ligation efficiency, the ChIP efficiency and the HiChIP library complexity were QCed following Arima’s guidelines (HiChIP User Guide for Mammalian Cells using Transcription Factors and Histones). Each library was first sequenced using an Illumina Miseq and the data was analyzed using MAPS ^90^ (Model-based analysis of PLAC-seq and HiChIP). The shallow sequencing QC metrics output by the Arima-MAPS pipeline was obtained to assess library quality for the degree of ChIP enrichment and capture of long-range chromatin interactions, and the QC information in turn was used to estimate the required depth of sequencing depth needed for robust and reproducible chromatin loop discovery (Arima-HiChIP Bioinformatics User Guide). Finally, barcoded HiChIP libraries were pooled and sequenced using an Illumina Novaseq, with one run on an S4 flowcell, generating ~10 billion paired-end reads with 150bp read length, for a coverage of 0.5-1 million read pairs per technical replicate, 1-1.5 billion read pairs per culture.

Positive: Peak region around the *ERBB3* promoter chr12: 56477200-56477650, should apply to H3K27AC, H3K4Me1 and H3K4Me3, but is negative for H3K27Me3 (Fpos and Rpos, **Supplementary Table 12)**

Negative: non-acetylation region in chromosome 17:chr17:51211300-51211500, good for negative ctrl for both H3K27Ac and H3K27Me3 (Fneg, Rneg, **Supplementary Table 12**)

### HiChIP data processing using HiC-Pro:

HiChIP sequencing data from the melanocyte cultures (3 replicates per culture) were processed using the HiC-Pro pipeline (version 3.1.0) ^91^, which provides a sequential integrated framework for read alignment, filtering, quality control, and generating data for valid chromatin interaction pairs. HiChIP sequencing reads were aligned to the hg19 human reference genome using Bowtie2 ^92^ in an iterative mode, to identify both standard and chimeric reads arising from proximity ligation. HiC-Pro uses a dual mapping strategy to first align individual read ends and then reconstruct valid ligation pairs based on restriction fragment information. Restriction fragment information was available from Arima Genomics. Reads with low mapping quality (MAPQ < 30), PCR duplicates, self-circularized fragments, dangling ends, other invalid ligation products, and PCR-associated duplicated valid pairs were filtered out. Only valid interaction pairs mapping to two different restriction fragments were retained for downstream analysis. HiC-Pro then assigned read pairs to restriction fragments using a predefined restriction digestion map and categorized interactions as *cis* (intra-chromosomal) or *trans* (inter-chromosomal). The valid interaction pairs were binned and assessed at multiple bin size resolutions 2.5 kb, 5 kb, and 10 kb to balance sensitivity in loop detection with computational efficiency. HiC-Pro internally corrects for biases such as coverage, restriction fragment length, and mappability via implementing the iterative correction and eigenvector decomposition (ICE) normalization ^93^. This normalization ensures uniform visibility of each fragment bin, making contact frequencies comparable across regions and replicates. Quality control metrics, including total and unique valid pair counts, proportions of *cis* versus *trans* interactions, long-range (>20 kb) interaction frequencies, and reproducibility between replicates, were used to assess library complexity and interaction capture efficiency and a summary of these for each technical replicate per culture is provided under **Supplementary Table 14**.

### FitHiChIP Loop Calling:

Significant chromatin contacts were identified using the FitHiChIP pipeline (version 11.0) ^94^, which detects contacts above a significance threshold while accounting for coverage and distance-dependent biases. The input consisted of all valid pairs identified by the HiC-Pro pipeline, binned at a 2.5 kb resolution, along with ChIP-Seq peak information corresponding to the immunoprecipitated H3K27ac antibody. Valid pairs from the technical replicates across each independent melanocyte culture were combined, and the three melanocyte cultures were treated as biological replicates for both chromatin interaction contacts and peak calling. ChIP peaks were called using the HiC-Pro HiChIP output using the FitHiChIP PeakInferHiChIP.sh utility, which incorporated MACS2 tool (version 2.2.7.1) ^95^ for calling H3K27ac significant peaks. Significant chromatin contacts were identified using a q-value threshold of 0.01. FitHiChIP was run in the IntType=3 configuration to detect both peak-to-peak and peak-to-nonpeak interactions. Because H3K27ac marks both enhancer and promoter elements ^96-98^, peak-to-peak chromatin contacts corresponded to potential promoter–enhancer, enhancer–enhancer, or promoter–promoter interactions. Interaction distances were defined within the default range of 20 kb to 2 Mb. Bias correction was performed using coverage-based regression (BiasType=1) to model the expected contact probability. Background modeling incorporated both anchor types (UseP2PBackgrnd=0), and merge filtering (MergeInt=1) was applied to consolidate proximal and redundant loop calls. All looping events were called at a 2.5 kb bin resolution using a false discovery rate (FDR) threshold of 0.01.

To extract significant loops connecting the deletion region to promoter regions, we used bedtools intersect tool ^81^. Promoter regions were defined based on chromHMM state annotations from melanocyte cells ^43,67,88^, using GENCODE version 19 protein-coding transcripts as described above. Since our deletion coordinates were in GRCh38, we converted HiChIP loops and promoter coordinates to GRCh38. The resulting significant loops were exported in long-range format for visualization on the WashU Epigenome Browser using GRCh38 coordinates. In the long-range interact output file, each reported position represents the midpoint of the fixed-size fragment bin. The score field denotes the negative log10 of the chromatin interaction q-value; if this value is less than or equal to zero, a default score of 500 was assigned.

### Loop Catalog H3K27Ac-HiChIP dataset processing:

we analyzed H3K27Ac-HiChIP datasets from Loop Catalog (https://loopcatalog.lji.org/loops/?genome=hg38) to assess chromatin interactions between the deletion region and gene promoters. Briefly, we downloaded the merged FH loops (FitHiChIP with peaks inferred from HiChIP) 5kb peak-to-peak files for all samples with high quality (assigned as Pass, *i.e.* QC scores from 8 to 10) ^45^. Due to the majority of Loop Catalog having lower sequencing coverage than our data, we considered the top 16 datasets with number of peak-to-peak loops in the top 20 percent (*i.e.* minimum of 90,014 peaks). Bedtools intersect ^81^ was used to extract loops located within the deletion region and promoters of genes in 9p21 (**Supplementary Table 9**).

### Predicting effects of the 9p21 deletion on gene activity:

we used AlphaGenome^46^ to assess the regulatory effects of the 9p21 deletion on nearby genes in different cell lines/tissues. Specifically, we compared the predicted functional genomics tracks between the wildtype (WT) and deletion alleles within a 524,288 bp genomic window upstream of the variant position using the “predict_sequence” function ^46^. Predict_sequence was performed on a reference sequence string consisting of full sequences (chr9:21,756,689-22,280,977; hg38) and an alternative sequence string with the deletion sequence being padded as Ns (chr9:21,756,689-22,180,210; hg38) to meet AlphaGenome genomic window (524,288 bp). Predictions were generated in several cell types and tissues, including foreskin fibroblasts (Cell Ontology: CL:1001608), foreskin keratinocytes (CL:1001606), foreskin melanocytes (CL:2000045), keratinocytes (CL:0000312), fibroblast of dermis (CL:0002551), melanocyte of skin (CL:1000458), melanoma cell line A375 (EFO:0002103), melanoma cell line SK-MEL-5 (EFO:0005720), skin of body (UBERON:0002097) and lower leg skin (UBERON:0004264). Due to the limitation of available assays for each cell type/tissue, not all assays are available for all cell lines/tissues selected. We assessed changes in transcription (RNA-seq) for all selected cell lines/tissues; while changes in chromatin accessibility (H3K4me3, H3K9ac, H3K27ac and H3K36me3 ChIP) were analyzed for the cell lines with available data. All analyses were performed on the human reference genome GRCh38. Deletion-associated changes in gene expression and regulatory activity were calculated as differences in predicted values (model-derived, normalized, unitless signal) of corresponding assays between the alternative and reference sequence across the locus.

### Tumor LOH analysis:

Using genotyped and imputed genotypes from genotyping array data ^58^, we selected 1,171 SNPs located on chromosome band 9p21 (from *MTAP* to *DMRTA1*) that are heterozygous across germline samples carrying the deletion (as well as other samples being assessed simultaneously). Due to a relatively low number of well-imputed SNPs over *CDKN2A* and *CDKN2B*, we manually added 70 additional SNPs (MAF >= 0.05 in the European population, 1000Genome project), bringing the total number of selected SNPs to 1,241 SNPs. For control regions, we selected 654 SNPs on chromosome 2 (313 SNPs), chromosome 19 (113 SNPs) and chromosome 21 (228 SNPs) respectively in regions that are infrequently subject to copy-number alterations in melanoma tumors ^55^. Five samples (20325-1001 Germline and Tumor, 6143-1001 Germline, 6143-2002 Germline and Tumor) were used in this LOH experiment. 20325-01001 was from a lymphonodal metastasis while 6143-02002 was a superficial spreading melanoma with very few melanoma cells and did not pass the QC, leaving a single tumor sample for the final analysis. Sequencing was performed in the same manner as described above for the Kapa HyperExome, except using a Roche Kapa HyperChoice capture kit with custom content. Design was performed using the Roche HyperDesign tool to target individual variants with 50bp padding on either side of each variant.

*S*equencing reads were aligned to the human genome assembly GRCh38 using the local alignment tool bwa mem ^76^, and variant calling was performed using bcftools mpileup followed by bcftools call function ^99^. We removed SNPs located on the deletion region (140 SNPs) from all downstream analysis since these SNPs are not informative. To account for sequencing bias due to differences in coverage among samples, we performed a QC step to select only SNPs with high coverage (minimum read counts of 50 reads) in all samples, 950 SNPs on 9p21 and 592 SNPs in control regions (chr2: 281 SNPs, chr19: 93 SNPs, chr21: 218 SNPs) passed this QC. SNP haplotype (*i.e.* SNP allele located on the same allele as the deletion) was identified as the allele shared by all three germline samples using both the imputed genotypes from the genotyping array and genotypes called by bcftools ^99^. In total, 651/950 SNPs have assigned haplotype, the remaining SNPs are either heterozygous (17 SNPs) or homozygous reference (281 SNPs) in all three germline samples or missing (1 SNP) in one of the samples (**Supplementary Table 10**).

Log R ratio (LRR) was calculated by taking the logarithm (base 2) of the ratio of total reads over the mean reads of all variants in the control regions. First, we assessed LRR of the control regions and found that the selected region on chr19 for 20325-1001_Tumor was deleted (**Supplementary Fig. 7**). Therefore, we excluded the SNPs on chr19 in LRR calculation for 9p21 for all samples. For SNPs on 9p21, haplotype allele frequency was calculated by the ratio of number of reads for the haplotype allele divided by total reads for each variant (**Supplementary Table 10**). LRR plots were generated using all SNPs that passed QC (950 SNPs) while Haplotype allele frequency plots were generated using only heterozygous SNPs in the germline samples (279 SNPs for 20325-1001_Germline) using the ggplot2 R package ^100^. Significant differences of LRR and Allele frequency between the germline and tumor samples were assessed by Paired T-test.

Further copy number analysis as well as estimation of tumor purity was performed using a Snakemake pipeline implementing CNV_FACETS ^48^ (https://github.com/dariober/cnv_facets), a command-line implementation of the FACETS algorithm optimized for targeted sequencing data. Samples were processed using its aligned BAM file, a VCF file containing common polymorphic SNPs, and a target-region BED file, all aligned to the GRCh38 reference genome. Because targeted sequencing panels contain sparse and unevenly distributed SNPs, CNV_FACETS parameters were tuned accordingly:

--cval 25 100 for pre-segmentation and final segmentation
--nbhd-snp 150 to reduce SNP-level serial correlation
- Default GC-correction and standard purity–ploidy grid-search settings

These parameter choices follow published recommendations for applying FACETS to targeted sequencing, where higher noise and lower SNP density require more permissive segmentation. Somatic copy number alterations (SCNAs) were identified by pairing each tumor sample with its patient-matched normal using the standard paired FACETS model. In paired mode:

- logR is computed as the tumor-to-normal depth ratio,
- logOR is computed as the difference in allelic odds ratio between tumor and normal, which cancels reference-mapping bias,
- FACETS infers segment-level TCN, major/minor allele copy numbers, LOH, and tumor purity and ploidy.

Segments showing copy-number changes in the tumor that were not present in the matched normal were classified as somatic SCNAs.

### Analysis of haplotype sharing across deletion carriers:

Samples were genotyped on one of three different array platforms (Illumina OmniExpress, Global Screening Array/GSAMD-24v2 or GSAv3Confluence) (**Supplementary Table 15**). We imputed genotypes for chromosome 9 variants separately for each platform. To minimize imputation error/artifacts caused by misgenotyping of variant positions within the deletion region, we excluded genotyped SNPs within this region prior to imputation. Imputation was performed using the TOPMed Imputation Server using TOPMed [version r3] as the imputation reference. The deletion samples were imputed along with an additional 500 samples from each array.

Amongst a set of individuals that share a common founder haplotype, at any given bi-allelic marker within the shared region these individuals can only be heterozygotes, or alternatively homozygous for the allele on the shared haplotype. Loss of haplotype sharing amongst these individuals is therefore indicated by stretches of multiple nearby variants each with homozygous genotype calls for both alleles. We therefore adopted the following strategy to scan deletion carriers for loss of sharing and define boundaries of shared haplotypes amongst deletion carriers:

1. We used data from unrelated individuals; where a family had multiple cases genotyped, we retained only one sample for analysis.
2. To minimize the potential for genotyping or imputation errors to confound the analysis, we filtered the data to retain only those variants with a MAF > 0.01 and INFO > 0.3 across each of the imputed datasets. We used best-guess genotypes, however, where genotype dosages showed uncertainty (deviating more than 0.1) or moving from the deletion towards the telomeric (or separately towards the centromeric) end of chromosome 9, we scanned for informative discordant positions containing homozygote genotypes for two different alleles amongst a set of unrelated deletion carriers.
3. Where there are multiple supporting discordant homozygote genotype calls within 50 Kb, we called a breakpoint at the discordant position nearest to the deletion. Where the discordant homozygous genotype for one sample differed from the homozygous genotype of multiple other samples, that sample was considered to be the one with a break in the founder haplotype; where markers were ambiguous (for example there was one sample homozygous for each of the two alleles) we scanned for additional less-ambiguous discordant markers to identify the sample with lost allele sharing.
4. Once loss of sharing was observed for an individual, the genotype data for that individual was removed from the datasets and the process was repeated iteratively, moving outward from the prior breakpoint in the same direction.
5. The final two samples are assigned the same breakpoint.

### Estimation of the age of the most recent common ancestor of carriers of the 9p21 deletion:

To estimate the number of generations since the most recent common ancestor (MRCA) of chromosomes carrying the 9p21 deletion, we first translated inferred shared-haplotype boundaries from physical coordinates to genetic coordinates. Physical breakpoint positions were mapped to sex-specific genetic maps from deCODE Genetics, using the corresponding chromosome 9 recombination map. For each inferred telomeric and centromeric breakpoint, local genetic map positions were obtained by linear interpolation between adjacent deCODE map markers, and the genetic distance from the deletion to the breakpoint was calculated in Morgans.

Let *n* denote the number of generations since the MRCA of all *deletion-bearing chromosomes*. We assume that (1) the deletion arose once in a founder *chromosome*, (2) deletion-bearing chromosomes segregate independently conditional on the founder event, and (3) the observed haplotype boundaries represent historical recombination events flanking the deletion. The method extends the likelihood formulation of Genin et al. ^49^ to phased SNP-based haplotypes.

Assume the haplotype segment of interest has a left-side boundary at recombination fraction *θ* from the deletion. Under a single-ancestor model, the probability that no recombination occurred at this locus over *n* generations is *S*(*θ*, *n*) = (1 − *θ*)^*n*^. Let f(θ,n) denote the probability that the first recombination breakpoint occurs exactly at *θ*. The implication is: (1) no recombination before *θ* − Δ*θ* and one recombination between *θ* − Δ*θ* and *θ*. So

$$f(\theta ,n)=-\underset{\Delta \theta \to 0}{\text{lim}}\frac{S(\theta -\Delta \theta ,n)-S(\theta ,n)}{\Delta \theta }=\frac{\partial S(\theta ,n)}{\partial \theta }=n{\left(1-\theta \right)}^{n-1}.$$

We first derive the log likelihood for left-side haplotype breakpoints. Let *N* denote the total number of carriers. Order the observed left recombination fractions as

$$\theta_{0}\leq \theta_{1}\leq \cdots \leq \theta_{N-2},$$

where *θ*_0_ corresponds to the longest shared haplotype observed among two individuals. We define group *G*_1_ consisting of two individuals sharing a haplotype of length *θ*_0_, and group *G*_2_ consisting of the remaining *N* − 2 individuals with breakpoints at *θ_k_* (*k* = 1,…, *N* − 2).

For subjects in group *G*_1_, one of the two individuals has the first recombination exactly at *θ*_0_; the other shares the ancestral haplotype beyond *θ*_0_. Thus, the likelihood is given as $2S(\theta_{0},n)f(\theta_{0},n)$. For subjects in group *G*_2_, the likelihood is calculated as $\prod_{k=1}^{K-2}f(\theta_{k},n)$. Thus, the total log likelihood for left-side breaks points is given as

$$l=\log\;2S(\theta_{0},n)f(\theta_{0},n)+\sum_{k=1}^{N-2}\log\;f(\theta_{k},n),$$

which simplifies as

$$l_{1}=(2n-1)\;\log(1-\theta_{0})+(N-1)\;\log\;n+(n-1)\sum_{k=1}^{N-2}\log(1-\theta_{k}).$$

The same argument applies to the right side with recombination fractions $\gamma_{0}\leq \gamma_{1}\leq \cdots \leq \gamma_{N-2}$:

$$l_{2}=(2n-1)\;\log(1-\gamma_{0})+(N-1)\;\log\;n+(n-1)\sum_{k=1}^{N-2}\log(1-\gamma_{k})$$

Maximizing the total log likelihood *l*_1_ + *l*_2_ with respect to *n* yields an estimator

$$\hat{n}=\frac{2(1-N)}{2\;\log(1-\theta_{0})+\sum_{k=1}^{N-2}\log(1-\theta_{k})+2\;\log(1-\gamma_{0})+\sum_{k=1}^{N-2}\log(1-\gamma_{k})}.$$

The second derivative of the log likelihood is $l^{\prime \prime }=-\frac{2(N-1)}{n^{2}}$; thus, we can derive the standard error as $se(\hat{n})=\hat{n}∕\sqrt{2(N-1)}$.

## Supplementary Material

Supplementary Files

This is a list of supplementary files associated with this preprint. Click to download.

- SupplementaryNoteFINAL.pdf
- SupplementaryTables8FINAL630PM.xlsx
- SupplementaryFiguressubmissionv8FINAL.pdf

Supplementary Information

1. **Supplementary Note:** Description of the MelaNostrum study cohorts

2. Supplementary Figures

Figure S1: Genotyping of the deletion using a multiplexed PCR assay

Figure S2: Flowchart summary of samples used for identification of additional carriers and association analysis

Figure S3: Pedigrees of 12 families with deletion

Figure S4: AlphaGenome prediction for effects of the 9p21 deletion on transcription

Figure S5: CTCF-binding and HiC interactions across 9p21

Figure S6: Principal components analysis displays similarity in genetic components of Genoa samples and other Italian controls

Figure S7: Log R Ratio (LRR) for SNPs in the control regions for tumor sequencing

3. Supplementary Tables (all in one excel file, each tab contains one table)

Table S1: Phenotypic characterization of all melanoma cases in families carrying deletion

Table S2: Common and rare structural variants called by ClinSV for 7646-01005 WGS

Table S3: Known structural variants called by DGV/gnomAD

Table S4: Summary of rare variants (MAF <1 x 10^−4^, gnomAD v.4, Non-Finnish European) for three 7646 family cases in the 9p21 locus (chr9:19,900,001-33,200,000; hg38) from WES data

Table S5: MelaNostrum WES study population

Table S6: List of all carriers of rs755147810, rs375317034 and deletion

Table S7: Fisher Exact tests for association of *MTAP* rs755147810 variant with melanoma risk

Table S8: Melanocyte H3K27Ac HiChIP data for peak-to-peak loops originating from deletion region

Table S9: Summary for the top 20 percent H3K27Ac HiChIP datasets from Loop Catalog

Table S10: Haplotype allele frequency and Log R Ratio of the 20325-01001 germline and tumor samples

Table S11: CNV_FACETS results for 20325-01001 Tumor

Table S12: Primer sequences

Table S13: ReMap 2022 CTCF ChIPseq non-redundant peaks located within the deletion

Table S14: Melanocyte H3K27Ac HiChIP sequencing statistics

Table S15: SNP array information for deletion carriers
